# Standardized protocol to detect phosphorylated α-synuclein in skin biopsies: recommendations from an expert panel

**DOI:** 10.1186/s13024-026-00966-w

**Published:** 2026-06-25

**Authors:** V. Donadio, C. Gibbons, C. Delprete, K. Doppler, A. Furia, A. Incensi, M. Ingelsson, J. Yang, M. Liu, G. Melli, M. Nolano, V. Provitera, G. Rizzo, K. Tsukita, R. Freeman, R. Liguori

**Affiliations:** 1 IRCCS Istituto delle Scienze Neurologiche di Bologna, UOC Clinica Neurologica, via Altura 3, 40139 Bologna, Italy; 2 Dipartimento di Scienze Biomediche e Neuromotorie, Università di Bologna, Bologna, Italy; 3 Department of Neurology, Beth Israel Deaconess Medical Center Boston, Boston, USA; 4 Department of Neurology, University Hospital Würzburg, Würzburg, Germany; 5 Department of Public Health and Caring Sciences, Geriatrics, Uppsala University, Uppsala, Sweden; 6 Krembil Brain Institute, University Health Network, Toronto, Ontario Canada; 7 Tanz Centre for Research in Neurodegenerative Diseases, Departments of Medicine and Laboratory Medicine & Pathobiology, University of Toronto, Toronto, Ontario Canada; 8 Department of Neurology, First Affiliated Hospital of Zhengzhou University, Zhengzhou, Henan China; 9 Institute for Translational Research (IRT), Faculty of Biomedical Sciences, Università della Svizzera italiana (USI) and Ente Ospedaliero Cantonale (EOC), Lugano, Switzerland; 10 Neurology Department, Neurocenter of Southern Switzerland, Ente Ospedaliero Cantonale, Lugano, Switzerland; 11 Department of Neurosciences, Reproductive Sciences and Odontostomatology, University Federico Il of Naples, Naples, Italy; 12 Neurology Department, Skin Biopsy Laboratory, Istituti Clinici Scientifici Maugeri IRCCS, Telese Terme, Italy; 13 Department of Neurology, Graduate School of Medicine, Kyoto University, Kyoto, Japan

**Keywords:** Skin biopsy, Phosphorylated α-synuclein at ser129, Biomarker, Standardization, Expert panel

## Abstract

**Background:**

Skin biopsy is a promising diagnostic tool for the in vivo identification of Parkinson’s disease and other α-synucleinopathies. However, despite increasing adoption, methodological variability exists among different laboratories applying this technique. To move toward a formal standardized protocol, leading experts in the field convened for a meeting focused on discussing and harmonizing the main methodological differences. The outcome of this meeting was a consensus-based skin biopsy protocol, which is here presented.

**Results:**

A formal expert opinion conference was held on November 15–16, 2024, at the IRCCS Institute of Neurological Sciences of Bologna (Italy). The consensus was reached by using a Question & Answer format following the PICO (Population, Intervention, Comparison, Outcome) methodology. An online Delphi survey was then performed based on the contrasting issues raised during the meeting. Unanimous consensus was reached on key elements, including the skin biopsy technique, choice of fixative, use of a cryostat, staining method for phosphorylated α-synuclein (p-α-syn), use of high-power fluorescence microscope, co-localization of p-α-syn with neuronal or glial markers, and high-magnification analysis. Additional agreement, achieved through the Delphi method, concerned specific procedural parameters such as the number of samples and section thickness, punch diameter, preferred biopsy sites in cases of asymmetric motor involvement, quantitative methods for assessing p-α-syn load, and use of p-α-syn as the optimal marker for identifying misfolded α-synuclein in the skin.

**Conclusions**

We have developed a consensus-based protocol for the detection of p-α-syn in skin samples from patients with suspected α-synucleinopathies, with the aim of promoting standardization, a broader adoption of the method, and its translation into clinical practice.

**Supplementary Information:**

The online version contains supplementary material available at https://doi.org/10.1186/s13024-026-00966-w.

## Background

The clinical diagnosis of Parkinson’s disease (PD) and other α-synucleinopathies can be difficult because of the clinical complexity of overlapping clinical phenotypes with non-synuclein disorders such as tauopathies or vascular diseases [1, 2]. A simple and reliable biomarker for these disorders is urgently needed to help identify diseases with different prognoses, to aid in clinical management and to monitor the effect of disease modifying therapies in clinical trials. Skin biopsy is a promising diagnostic tool for α-synucleinopathies because it enables detection of misfolded or phosphorylated α-synuclein (p-α-syn) deposits with high specificity and sensitivity compared to healthy controls [3, 4, 5, 6, 7, 8, 9, 10]. This technique is reliable, repeatable, cost-effective, safe and easy-to-perform even in resource limited environments and is associated with only minor discomfort for the patient. With this approach skin p-α-syn deposits have been shown to potentially differentiate all variants of α-synucleinopathies [11, 12, 13, 14, 15] and to identify also prodromal stages such as idiopathic rapid eye movement sleep behavior disorder (iRBD) [16, 17, 18]. 

However, the processing methodologies may create challenges to reproducibility. The variability in laboratory techniques includes diameter of the biopsy, body location from which the biopsies are taken, total number of biopsy samples, thickness of skin sections, selection of primary antibody, signal amplification method, and the quantification method to estimate the p-α-syn load. To overcome these methodological differences, we propose the adoption of a standardized protocol across the various laboratories. This would allow for the generation of comparable data, between the different laboratories analyzing skin p-α-syn, which would support the clinical application and large scale adoption of this as a diagnostic method in patients with suspected α-synucleinopathies.

To achieve this goal, leading experts in the field convened for a meeting focused on discussing the main methodological aspects that differ across laboratories. The meeting resulted in a shared decision on a skin biopsy protocol, which is outlined in this report. Furthermore, during the meeting the main current data on the diagnostic accuracy of skin biopsy in the identification of different α-synucleinopathies were summarized.

## Methods

A formal expert opinion conference was held on November 15th and 16th, 2024, at the IRCCS Institute of Neurological Sciences of Bologna (Italy). Several experts from Europe, Asia, and America attended the meeting. The participants were physicians (neurologists and geriatricians), biologists, and laboratory technicians with experience of skin p-α-syn search. The main selection criterion for the experts was proven experience with immunofluorescence for the detection of phosphorylated alpha-synuclein in the skin, as demonstrated by the publication of at least 2 papers or 1 meta-analysis. They included Alessandro Furia (Bologna, Italy), Alex Incensi (Bologna, Italy), Cecilia Delprete (Bologna, Italy), Chris Gibbons (Boston, USA), Giorgia Melli (Lugano, Switzerland), Giovanni Rizzo (Bologna, Italy), Jing Yang (Zhengzhou, China), Kazuto Tsukita (Kyoto, Japan), Maria Nolano (Napoli and Benevento, Italy), Martin Ingelsson (Toronto, Canada and Uppsala, Sweden), Minglei Liu (Zhengzhou, China), Rocco Liguori (Bologna, Italy), Roy Freeman (Boston, USA), Vincenzo Donadio (Bologna, Italy) and Vincenzo Provitera (Benevento, Italy). Kathrin Doppler (Würzburg, Germany) attended the meeting remotely through an online connection.

The expert panel’s activities were organized into the following methodologically distinct phases, all of which were approved by the participants:

- ***Phase I: before the meeting***. In the **scoping phase** the experts defined the protocol for literature review and systematic research, as well as topics to be discussed during the meeting. In the **assessment phase** a systematic review of the literature on the application of skin biopsy to detect α-syn was performed. This review was reported according to the PRISMA guidelines [19]. Criteria for study selection and consideration were: detection of skin pathological α-syn by indirect immunofluorescence co-localized with a neuronal or glial marker; original studies; case-control design; accepted clinical diagnostic criteria for α-synucleinopathies [20, 21, 22, 23]. The considered outcome measures included: diagnostic performance parameters, reliability parameters, diagnostic accuracy parameters. Reviews, position papers, case reports and and studies on animals were not considered. Published studies were identified from: (a) the National Library of Medicine’s PUBMED database, and (b) Elsevier’s SCOPUS platform; the search strategies included the use of the following syntax: skin biopsy OR skin innervation OR skin nerves OR skin samples AND alpha synuclein, native alpha synuclein, oligomer alpha synuclein, phosphorylated alpha synuclein, synucleinopathy, PD, MSA, RBD, DLB, PAF. Reference lists of identified articles were reviewed to find additional references. Two authors independently assessed studies, and any disagreement was planned to be solved by consensus with a third author. The main original data were thus revised, and a Conference Procedures (CP) draft was prepared. The draft was created as a Question & Answer document using a PICO format [24, 25] as specified in Suppl. Fig. 1. The draft listed all possible methodological options related to the procedures to collect and analyze skin samples for the p-α-syn detection by skin biopsy to be discussed during the Bologna meeting;
- **Phase II: live meeting. **The experts discussed literature-based aspects and shared their own specific laboratory experiences. For each aspect, all the options available from the scientific literature were analyzed. On some aspects, all participants agreed and expressed unanimous consensus, while on others, differing views emerged. For these latter aspects, the various options were selected and included in the Delphi survey. Selection of options to be included in the Delphi survey was reviewed and agreed upon by all participants. Furthermore, the clinical application of the skin biopsy in cases with suspected α-synucleinopathies was also analyzed to develop shared indications on the use of biopsy in each individual variant of α-synucleinopathy; 
- **Phase III: online Delphi survey.** Based on the choices made during the live meeting, a survey was sent to all participants regarding the main issues related to procedures for analyzing skin samples for p-α-syn detection, with the aim of reaching an agreement through a Delphi method.

### Delphi survey

The online survey was developed based on the issues raised during the in-person meeting where unanimous agreement among the panel was not reached (Table 1).

**Table 1:** Table 1. Questions for the online Delphi survey based on the issues raised during the in-person meeting where unanimous agreement was not reached among the panel.

Question 1	Question 2
Do you agree that the following two methods have similar diagnostic accuracy searching for m-α-syn: • Method 1: A biopsy of 3 skin sites (cervical, thigh and leg) with 1 sample for each skin site and analysis at 50 microns of 6 skin sections for each sample • Method 2: A biopsy of 2 skin sites (cervical and leg), with 2 samples for each site and analysis at 10 microns of 3 skin sections for each sample	What do you consider to be the optimal diameter of the punch, taking into account its advantages and disadvantages, for performing skin biopsies aimed at detecting abnormal deposits of α-synuclein?
Yes	3 mm
No	5 mm
I don’t have the expertise to answer this question	I don’t have the expertise to answer this question
**Question 3**	**Question 4**
In the case of a patient with Parkinson’s Disease (PD) and asymmetric symptoms, which side of the body would you consider optimal for a biopsy to detect abnormal accumulations of m-α-syn?	Is the Distribution Coefficient (DC) or similar methods to describe the distribution pattern of p-α-syn helpful in differentiating PD from other α-synucleinopathies?
Symptomatic site	Yes
Asymptomatic site	No
I don’t have the expertise to answer this question	I don’t have the expertise to answer this question
**Question 5**	**Question 6**
Are the p-α-syn load, fibers rate and number of fibers methods useful to express clinical involvement in α-synucleinopathies?	Is the p-α-syn S129 currently the best marker to identify misfolded α-syn in skin tissue?
Yes	Yes
No	No
I don’t have the expertise to answer this question	I don’t have the expertise to answer this question

The techniques requiring additional review prior to consensus included:

1. methods of analyzing skin samples for p-α-syn detection and analysis:
  - the number of samples to be used and.
  - the thickness of the skin sections to be analyzed;
2. diameter of the punch biopsy tool to be employed;
3. biopsy sampling sites in cases of asymmetric motor manifestations;
4. quantitative methods for assessing p-α-syn load;
5. assessment of p-α-syn as the optimal marker for misfolded/aggregates α-synuclein in the skin.

For each specific question, the consensus among participants was defined by considering a threshold of 75% of agreement of the responses provided [26, 27]. 

**Ethics and consent to participate declarations**

not applicable.

## Results

The systematic review of the literature yielded 32 original studies for consideration [3, 4, 5, 6, 7, 8, 9, 10, 11, 12, 13, 14, 15, 16, 17, 18, 28, 29, 30, 31, 34, 36, 37, 38, 39, 40, 41, 42, 43, 44, 45], as summarized in the flowchart reported in suppl. Fig. 2.

During the meeting all procedural aspects of skin biopsy focused on the detection of p-α-syn were analyzed and discussed. Some of these procedures were followed by all participants and represent those on which unanimous consensus among experts was reached. Conversely, other procedures are performed differently across laboratories, and for these it was necessary to reach consensus through the Delphi method. The Delphi questionnaire was constructed with two possible answers for each question in relation to the main options based on data from scientific literature. In addition, the answer “I don’t have the expertise to answer this question” was provided for each question in case the participant did not feel able to answer based on their skills and/or scientific knowledge for the specific topic. In addition, all participants agree that skin biopsy offers the advantages of relatively low cost, ease of execution, and the ability to be repeated in long-term studies.

### Agreed aspects of the skin biopsy procedure

#### Contraindications to perform a skin biopsy and possible side effects

Skin biopsy has no absolute contraindications, except in the presence of skin lesions, such as dermatological conditions or scarring, that may compromise the reliability of nerve fiber analysis, or in cases of significant bleeding risk. The latter includes patients taking two different antiplatelet agents, a combination of an antiplatelet and an anticoagulant, or those with a thrombophilic disorder who are also on antiplatelet therapy.

The procedure is generally safe and well-tolerated, with minimal side effects, primarily minor bleeding and, in rare cases, local infection, with only minor discomfort for the patient [28]. 

#### How to perform a skin biopsy

Skin biopsy for the detection of p-α-syn accumulations should be performed on hairy skin, while glabrous skin (commonly sampled from a finger) is not recommended for this purpose, as it is significantly more painful without offering improved diagnostic accuracy for p-α-syn detection [4]. To minimize bleeding during the biopsy, a combination of a local anesthetic and epinephrine can be injected at the biopsy site. The epinephrine induces vasoconstriction, thereby reducing local blood flow and hemorrhage. The biopsy should ideally include a hair follicle at its center, as this area is rich in autonomic skin annexes, such as the arrector pili muscle, cutaneous blood vessels, and sweat glands, providing the highest likelihood of detecting p-α-syn deposits, which are primarily found in autonomic cutaneous nerves in conditions such as PD, DLB, and PAF.

There was no consensus among the meeting participants regarding the specific hairy skin sites to be biopsied; therefore, this issue was submitted to the Delphi survey and is addressed in the following section.

#### Fixative

The skin samples can be fixed in cold 4% paraformaldehyde or Zamboni’s fixative (4% paraformaldehyde plus picric acid) or PLP 2% (paraformaldehyde, lysine and periodate). The fixation time may range from 30 min at room temperature [12] to overnight at 4 °C (approximately 18 h), without resulting in significant differences in staining outcomes [5, 6, 8, 13]. Although a direct comparison between the two fixation protocols is still lacking, a study involving two European laboratories (Würzburg, Germany, and Bologna, Italy) indicated that the procedures yielded comparable results. Specifically, no differences were observed in intraneural p-α-syn staining, and both methods were deemed suitable for indirect immunofluorescence analysis of p-α-syn [29]. However, a prospective multicenter study directly comparing these different protocols in terms of the fixative used (paraformaldehyde vs. Zamboni vs. PLP) and fixation time (30 min vs. 18 h) is needed to establish the equivalence of these procedures.

#### Cryostat

The skin sections are cut while frozen, using either a cryostat set between − 20 °C and − 30 °C, or a freezing slide microtome at -20 °C.

#### Technique to stain p-α-syn

The preferred immunohistochemistry (IHC) technique for detecting phosphorylated α-synuclein (p-α-syn) in skin samples is indirect immunofluorescence (iIF). This method relies on the use of a primary antibody targeting p-α-syn, followed by a secondary antibody conjugated to fluorophores such as cyanine dyes or Alexa Fluor^®^ dyes, allowing visualization through fluorescence microscopy equipped with the appropriate filter sets.

The advantages of this technique include the ability to detect multiple signals within the same merged image, which enables co-localization studies, and the use of a fixation process that avoids the over-fixation commonly associated with conventional IHC performed with bright-field microscopy. Over-fixation is not recommended, as it may reduce the immunoreactivity and compromise staining quality [30]. 

#### Power lamp of the fluorescence microscope

The fluorescence microscope light source should be set to a power level sufficient to optimally visualize the fluorescent signal corresponding to p-α-syn deposits. It is generally recommended that the light source have a power output greater than 100 W.

#### P-α-syn must be co-localized with a neuronal or glial marker

As fluorescent signals in skin sections may occasionally arise from background noise or nonspecific staining due to fluorophore precipitates, we recommend considering a staining as positive for p-α-syn only when it is co-localized with a neuronal marker (typically the Protein Gene Product 9.5- PGP 9.5) or a marker of cutaneous non-myelinating glial cells (e.g., Neural Cell Adhesion Molecule -NCAM).

#### High magnification analysis

Since p-α-syn deposits are generally small in size, to appropriately define a positive staining and ensure neuronal or glial co-localization, it is advisable to perform the analysis using a microscope set with a ×40 plan apochromat objective.

#### Classification of a patient positive for p-α-syn

Considering the high specificity reported for p-α-syn staining [4, 6, 9, 10], a single skin section positive for p-α-syn deposits is considered sufficient to classify the patient as positive for α-synucleinopathy. There is not enough experience to determine the specificity of other methods that detect different pathological forms of α-syn, such as the aggregated species (5G4), oligomeric species (ASyO4), truncated species (syn105) forms [31], or tyrosine 125 phosphorylated α-syn (pY-syn), advanced glycation end products (AGEs), or ubiquitin [32]. 

### Skin biopsy procedures on which an agreement was reached by Delphi survey (Table 1; Fig. 1)

**Fig. 1:**
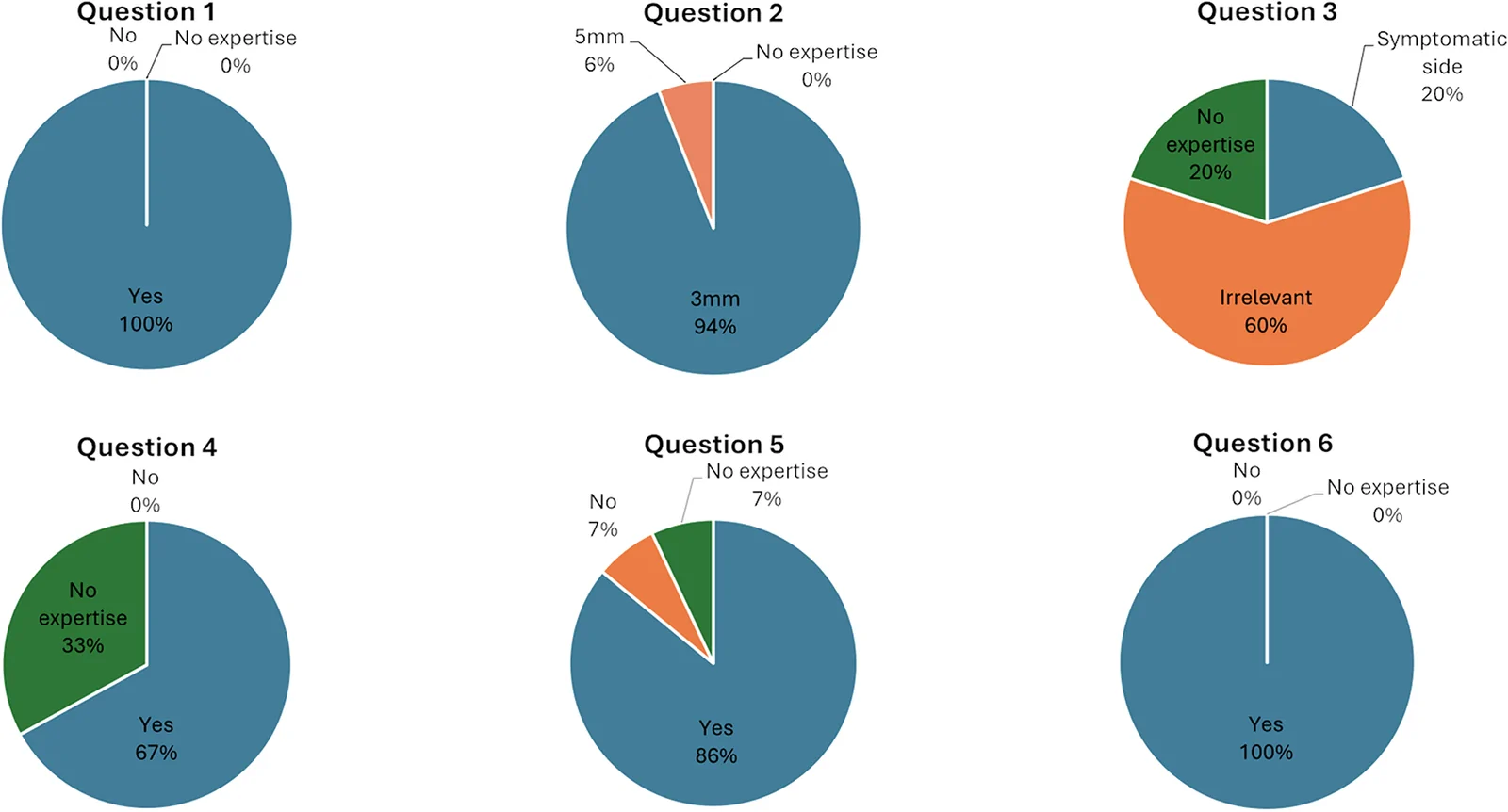
Survey results The survey was developed based on the issues raised during the in-person meeting where unanimous agreement among the panel was not reached. It was built around the following questions: the methods of analyzing skin samples for p-α-syn detection, considering the number of samples to be used and the thickness of the skin sections to be analyzed; the diameter of the punch to be employed; the sampling sites in cases of asymmetric motor disturbance; the quantitative methods for assessing p-α-syn load; and p-α-syn as the optimal marker for misfolded α-synuclein in the skin. The consensus among participants was defined by considering a threshold of 75% of agreement of the responses provided. Consensus was not reached for questions 3 (Sampling sites in asymmetric motor involvement) and 4 (Distribution Coefficient as a quantitative method for p-α-syn load)

#### Number of samples and section’s thickness (question 1)

By analyzing the available literature, it became evident that the number of biopsy sites and skin samples were closely related to the number of sections analyzed for each sample and the thickness of these sections. To address this, we summarized these aspects in the survey question by categorizing the procedures into Method 1 and Method 2 for detecting p-α-syn. **Method 1** involves performing the biopsy at three different sites (cervical, thigh, and leg), with one sample from each site, and analyzing six 50-micron sections from each sample. The cervical biopsy is performed at the C7 level, 5 cm lateral to the midline of the spine; the biopsy from the lateral thigh is obtained 10–15 cm proximal to the patella; and the leg biopsy is performed 10–15 cm proximal to the lateral malleolus. Samples with such a high thickness require free-floating analysis, which facilitates antibody penetration. The disadvantages of this approach are that it requires a skilled operator with experience in the manual analysis of skin sections and is time-consuming (approximately 3 days) [6, 8, 9, 12]. However, this method permits assessment of disorders with a distal to proximal gradient, e.g., small fiber pathology and also a comprehensive assessment of the topography of α-syn deposition. **Method 2**, on the other hand, requires biopsies from two skin sites (cervical and leg), with two samples from each site, and analyzing three 10-micron sections per sample. These thin samples can be analyzed directly on slides rather than in free-floating conditions. This procedure is faster than free-floating processing (approximately 2 days) and requires less manual expertise in handling skin sections, although the volume of skin sample analyzed is smaller [5, 7, 10, 11]. The higher number of samples to be analyzed using Method 2 (4 samples) compared with Method 1 (3 samples) is due to the need to compensate for the lower probability of detecting abnormal α-syn deposits in thin samples (10 μm of Method 2) versus thicker samples (50 μm of Method 1) due to the different skin tissue volume, as previously demonstrated [36]. The survey question asked whether the two methods had the same diagnostic accuracy in identifying p-α-syn accumulations. Upon reviewing the literature, all participants responded affirmatively, agreeing that both methods are equally effective in terms of diagnostic accuracy (100% “yes” response rate).

#### Punch diameter (question 2)

Based on the experiences reported in the literature, two different punch diameters have been used for detecting p-α-syn in the skin: 3 mm [3, 5, 6, 7, 8, 9, 10, 11] and 5 mm [4, 12, 16, 31]. The smaller punch diameter reduces the risk of bleeding and local infections, which are exceptionally rare with 3 mm punches [28] although no studies reporting complications associated with 5 mm punch diameter or directly comparing the two diameters are currently available. Consensus was reached on the use of the 3 mm punch as the optimal choice for this purpose, with 94% of responses favoring the 3 mm punch and 6% favoring the 5 mm punch.

#### Sampling sites in asymmetric motor involvement (question 3)

In patients with evident motor asymmetry, such as in early-stage PD, a key question arises regarding which side should be biopsied to search for abnormal α-syn. While the majority of participants (60%) agreed that p-α-syn should be sought in the skin regardless of the side of motor involvement, consensus was not reached on this issue. 20% of participants indicated that they lacked sufficient knowledge to answer the question, while another 20% believed the biopsy should be performed on the symptomatic side. The lack of agreement is likely due to the limited data available on this topic. However, the high sensitivity of p-α-syn across all studies performed without considering the symptomatic side probably suggests that the symptomatic side is not relevant for p-α-syn.

#### Quantitative methods for p-α-syn load (questions 4 and 5)

Among the quantitative methods for evaluating synuclein load reported in the literature, the most commonly used include the distribution coefficient (DC), which calculates the location of p-syn across the various skin sites biopsied as previously described; [6] p-α-syn load, which defines the number of annexes (i.e. nerve bundles, sweat glands, erector pilorum muscle, arterioles, hair bulb or sebaceous gland) that show positive p-syn fibers divided by the total number of annexes in all skin samples analysed; [15] fiber rate, rating the number of p-syn positive nerve fibers divided by the total number of annexes in all skin samples analysed; [33] and the number of fibers, which includes the absolute number of positive p-syn positive fibers [3]. Regarding the DC, it has been proposed as a useful tool to differentiate PD from other α-synucleinopathies [6]. The majority of participants (67%) consider this tool helpful, although the final result did not reach statistical significance for agreement, as 33% of participants felt they lacked sufficient knowledge to express a definitive opinion on the matter.

For the other quantitative methods, a consensus was reached, with 86% of participants agreeing that p-α-syn load, fiber rate, and the number of fibers are valuable methods for assessing clinical involvement in α-synucleinopathies. Regarding the overall topic of quantitative methods for p-α-syn, participants expressed the belief that current methods are somewhat approximate. They emphasized the need for future efforts to improve the quantification of p-α-syn accumulation, particularly through digital and automated techniques, including the use of artificial intelligence.

#### P-α-syn as the optimal marker for misfolded α-synuclein in the skin (question 6)

All participants agreed that the most reliable marker for detecting misfolded synuclein is phosphorylation at serine 129, due to its demonstrated reliability in numerous published studies. The highest yield has been observed with monoclonal antibodies derived from either rabbit or mouse, as reported in the literature [12, 33, 34], while polyclonal antibodies often lead to non-specific signals. Among the main anti-p-syn antibodies used with greater experience and showing the best diagnostic efficiency, we should include mouse monoclonal Covance, New Jersey, cat. no. MMS-5091 (1:500); [30, 38] mouse monoclonal Merck, cat. no. MAB826 (1:4000); [31, 34] rabbit monoclonal Abcam, Cambridge, UK, cat. no. ab51253 (1:2000); [31, 33, 34, 35] and rabbit monoclonal Cell Signaling, cat. no. 23,706 (1:2000) [34]. The primary staining can be enhanced by using proteinase K (PK) treatment [34], or it can be visualized by using more complex antigen retrieval techniques, such as formic acid or citrate buffer, in combination with biotin-streptavidin amplification [6, 36]. According to published reports, these two methods are equally effective in detecting p-α-syn [6, 34, 36]. 

### The diagnostic role of skin biopsy for each specific variant of α-synucleinopathies

During the meeting, data on skin biopsy in the different α-synucleinopathies reported in the medical literature were analyzed to assess how this technology can assist in differentiating these disorders in real-world clinical settings. Both skin innervation and p-α-syn deposits were considered. It was emphasized that the search for p-α-syn demonstrates high specificity, not only when comparing patients with α-synucleinopathies to healthy controls (96.6% to 99%) [6, 10], but also when differentiating patients with a confirmed diagnosis of other conditions (e.g., vascular diseases, tauopathies, Alzheimer’s disease, etc.), with specificity approaching nearly 100% [8, 11, 13, 15, 37]. The main differences observed across the α-synucleinopathies, which include the distribution pattern of p-α-syn deposition and cutaneous nerve degenerations, are as follows:

1. ***PD.*** P-α-syn deposition distribution may vary according to the disease stage, e.g., in fully established disease a proximal to distal gradient whereas in some prodromal patients, distal deposition is more prominent [6, 9, 11, 38, 39]. Autonomic fibers are more affected than somatic fibers [3, 4, 6, 11, 14, 31, 40]. Among the autonomic fibers, the adrenergic fibers of the cutaneous vessels are most affected; [4, 9, 11, 31, 32]
2. ***DLB.*** Some studies report a p-α-syn distribution similar to established PD whereas other report a more diffuse distribution [6, 13]. Small nerve fiber denervation is more severe than in PD [6] and autonomic fibers are more affected than somatic fibers [6, 13, 32] with equal involvement of adrenergic and cholinergic fibers; [13]
3. ***MSA.*** P-α-syn deposits have a widespread and diffuse distribution between distal and proximal sites but somatic fibers are more affected than autonomic fibers [6, 7, 9, 12, 14, 32, 39, 40, 41]. P-α-syn deposition in glial cells seems a more promising specific marker for MSA [7] although more data needs to be collected and an automated analysis program must be implemented. Contrasting findings were described about small nerve fiber denervation, some described nerve fiber loss to be less [6] or more [9] severe that seen in PD;
4. ***PAF.*** P-α-syn deposits present with a widespread and diffuse distribution between distal and. proximal sites with very high load [41]. Autonomic fibers are more affected than somatic fibers [5, 6] with a widespread involvement (i.e. adrenergic and cholinergic fibers) [5]. Small nerve fiber pathology is more severe than PD [6].
5. iRBD. P-α-syn shows a variable distribution, possibly reflecting that this condition may precede different α-synucleinopathies such as PD, DLB, or MSA. However, the rate of p-α-syn positivity is reported to range from 56% to 93% of studied patients, and remains consistent across all studies [28, 17, 18, 42–44]. No data are available regarding small fiber pathology.

#### Quantitative assessment of p-α-syn deposition

This analysis has revealed differences across the α-synucleinopathies with MSA > DLB> PD [6, 38]. In combination with quantitative assessment of p-α-syn, the topographic deposition, the selective involvement of somatic or autonomic nerve fibers and associated degeneration of small nerve fibers has shown a high degree of separation between MSA and PD, with > 90% of cases correctly identified in one multicenter study based on these pathologic findings [38]. 

#### Selection of healthy controls

The selection of control subjects for studies focused on the detection of cutaneous p-α-syn requires particular attention, as in the early stages synucleinopathies may present subtly, with symptoms that often go unnoticed. Therefore, in subjects selected as controls, the presence of an early extrapyramidal disorder, mild cognitive impairment (MCI), and subclinical autonomic dysfunction should be excluded.

Accordingly, in all subjects selected as the control group for comparative studies with patients affected by synucleinopathies, neurological deficits should be ruled out through a neurological examination, including assessment of olfactory dysfunction, together with administration of the following questionnaires: RBDSQ [33], to exclude a sleep disorder such as a RBD, COMPASS-31 [33], to exclude autonomic dysfunction, and MMSE and MoCA, to exclude cognitive impairment.

## Discussion

Alpha-synucleinopathies represent diseases of significant societal impact due to their high incidence, as in the case of PD and DLB, but also because of the severe disabilities associated with their clinical course, such as in MSA, DLB and PAF. Furthermore, several new disease-modifying therapeutic approaches have been developed and are currently undergoing clinical trials for these conditions. In this context, having a reliable and specific biomarker for these diseases could improve the clinical course of patients through targeted and appropriate treatment. It could also facilitate a more uniform selection of patients for specific clinical and therapeutic trials, decrease sample size, and support a more rigorous assessment of any disease-modifying treatments.

Skin biopsy for the detection of misfolded α-synuclein has proven to be a useful biomarker for the α-synucleinopathies [48, 49]. However, the variability in protocols used across different laboratories has created challenges in unifying clinical data sets and has created impediments to widespread adoption in research and clinical practice.

To address this, we convened a meeting with leading international experts in the immunohistochemical detection of cutaneous p-α-syn by means of skin biopsy with the aim of establishing and disseminating a standardized laboratory protocol for use in the α-synucleinopathies. We have outlined recommendations on all aspects of the procedure, which includes both the execution of the skin biopsy and its analysis to detect p-α-syn. The protocol includes aspects that were agreed upon by all participants, as well as those for which differences emerged between participants, necessitating consensus through a Delphi survey.

**Fig. 2:**
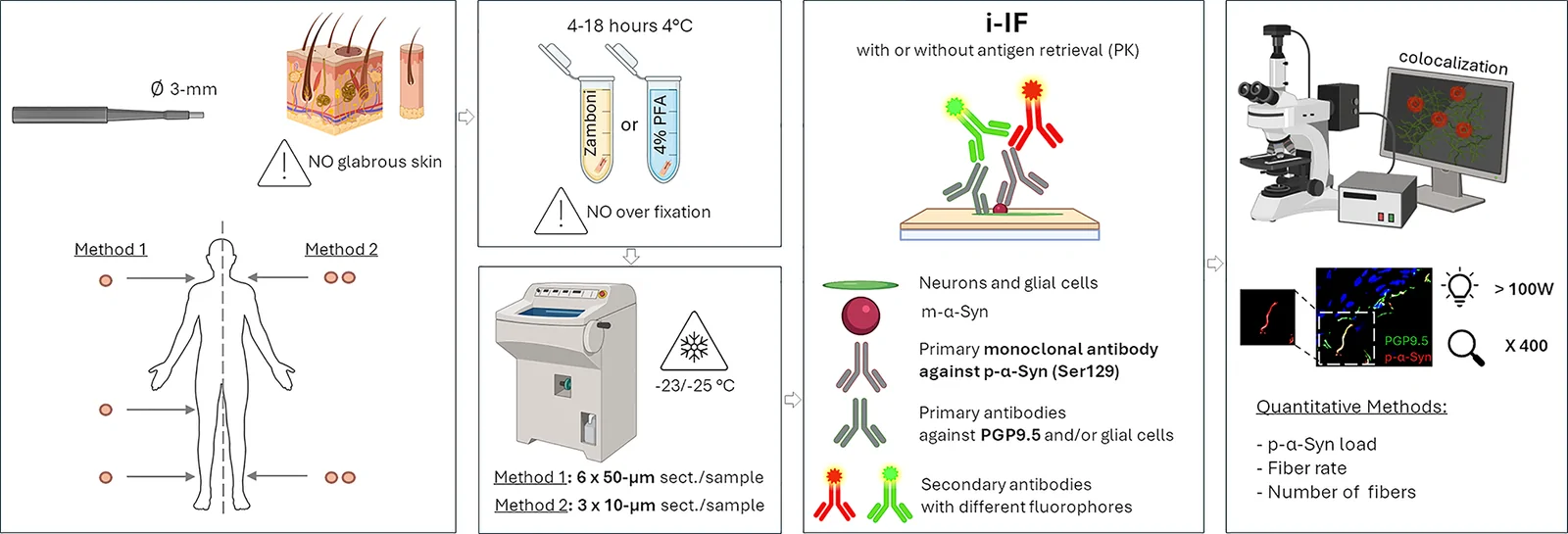
Skin biopsy protocol searching for p-α-syn The figure outlines all the steps of the method, from skin sample collection to its analysis through indirect immunofluorescence, culminating in the visualization and quantification of abnormal p-α-syn deposits (see the Results section for further details)

At the conclusion of the discussions and Delphi survey, a protocol was developed that will enable the use of a uniform method across laboratories employing this technique, improving the standardization of the methodology. The shared protocol that we therefore propose to adopt is summarized in Fig. 2. The figure outlines all the steps of the method, from skin sample collection to its analysis through indirect immunofluorescence, culminating in the visualization and quantification of abnormal p-α-syn deposits.

Furthermore, we present the main findings obtained through skin biopsy for the detection of p-α-syn in the different variants of α-synucleinopathy. These data may support the use of this tool to differentiate the various clinical phenotypes of α-synucleinopathies based on the distinct distribution of p-α-syn and the assessment of skin innervation. The use of skin biopsy is thus particularly relevant for defining how its data can be leveraged to enhance the diagnostic accuracy of this technique in PD, DLB, MSA, PAF and iRBD. However, it should be emphasized that all these studies were based on a clinical definition of the disease, which may entail a certain degree of diagnostic uncertainty [48–50], whereas it would be more appropriate to assess the diagnostic performance of p-α-syn in a cohort of pathologically defined cases. The lack of autopsy data to determine the diagnostic accuracy of p-α-syn in synucleinopathies represents a limitation of these studies that will need to be addressed in future research.

## Supplementary Information

Below is the link to the electronic supplementary material.

Supplementary Material 1

Supplementary Material 2

Supplementary Material 3

## Data Availability

The datasets generated and analysed during the current study are available from the corresponding authors on request.

## Abbreviations

PICO: Population Intervention Comparison Outcome
α-syn: α-Synuclein
p-α-syn: Phosphorylated α-synuclein at serine 129
PD: Parkinson’s disease
iRBD: Idiopathic rapid eye movement sleep behavior disorder
MSA: Multiple system atrophy
DLB: Dementia with Lewy bodies
PAF: Pure autonomic failure
CP: Conference Procedures
IHC: Immunohistochemistry
iIF: Indirect immunofluorescence
PGP 9.5: Protein Gene Product 9.5
NCAM: Neural Cell Adhesion Molecule
pY-syn: Tyrosine 125 phosphorylated α-syn
AGEs: Advanced glycation end products
DC: Distribution coefficient
PK: Proteinase K
