# Supplementary material for: Standardized protocol to detect phosphorylated α-synuclein in skin biopsies: recommendations from an expert panel
Source: Mol Neurodegener. 2026 Jun 25;21:54. doi: 10.1186/s13024-026-00966-w (PMC13560214; doi:10.1186/s13024-026-00966-w)
Supplement: Supplementary file 1 [file 13024_2026_966_MOESM1_ESM.docx]

**Supplemental Fig. 1. Research topics and questions based on PICO**

**TOPIC 1: SKIN SITES TO BE ANALYSED (INCL. NUMBER AND TYPE OF SITES)**

*1.1 Which sites should be sampled?*

- Possible skin sites: e.g. cervical C7, thigh, leg, other

*In patients with suspected synucleinopathy, does indirect immunofluorescence in skin biopsy samples shows better diagnostic accuracy in specific body regions (e.g., leg, thigh, cervical) in detecting p-syn?*

| P | Patients with suspected or confirmed synucleinopathy (i.e. Parkinson’s disease, Multiple System Atrophy, Dementia with Lewy bodies, Pure Autonomic Failure), or healthy controls. |
| --- | --- |
| I/C | indirect immunofluorescence in skin biopsy samples in different body sites (cervical C7, thigh, leg, other) |
| O | Frequency of analysable specimens  Diagnostic accuracy indicators in detecting p-syn: sensitivity, specificity, overall accuracy, false rates  Reliability indicators: raw agreement, kappa statistic |
| Study design | - consecutive suspected patients  - patients vs healthy controls |

*1.2 How many sites should be collected?*

*-* Possible number of skin sites: e.g. 1 vs 2 vs 3 vs 4 sites?

*In patients with suspected synucleinopathy does indirect immunofluorescence in skin biopsy samples, performs better diagnostic accuracy by collecting multiple samples compared to 1 in detecting p-syn?*

| P | Patients with suspected or confirmed synucleinopathy (i.e. Parkinson’s disease, Multiple System Atrophy, Dementia with Lewy bodies, Pure Autonomic Failure), or healthy controls. |
| --- | --- |
| I/C | indirect immunofluorescence in skin biopsy samples collecting multiple samples compared to 1 |
| O | Frequency of analysable specimens  Diagnostic accuracy indicators in detecting p-syn: sensitivity, specificity, overall accuracy, false rates  Reliability indicators in detecting p-syn: raw agreement, kappa statistic |
| Study design | - consecutive suspected patients  - patients vs healthy controls |

*1.3 Should collection be unilateral or bilateral or symptom-oriented?*

*-* Mono vs bilateral vs most affected side

*In patients with suspected synucleinopathy, does indirect immunofluorescence in skin biopsy samples performs better diagnostic accuracy by collecting samples from unilateral site compared to bilateral or symptom-associated regions in detecting p-syn?*

| P | Patients with suspected or confirmed synucleinopathy (i.e. Parkinson’s disease, Multiple System Atrophy, Dementia with Lewy bodies, Pure Autonomic Failure), or healthy controls. |
| --- | --- |
| I/C | indirect immunofluorescence in skin biopsy samples collecting samples from unilateral site compared to bilateral or symptom-associated regions |
| O | Frequency of analysable specimens  Diagnostic accuracy indicators in detecting p-syn: sensitivity, specificity, overall accuracy, false rates  Reliability indicators in detecting p-syn: raw agreement, kappa statistic |
| Study design | - consecutive suspected patients  - patients vs healthy controls |

**TOPIC 2: NUMBER OF SKIN SAMPLES ANALYSED**

*2.1 How many samples should be analysed for each skin site region?*

- Possible number of samples: e.g., 1 vs 2 samples

*In patients with suspected synucleinopathy, does analysis of indirect immunofluorescence for phosphorylated synuclein (p-syn) in skin samples at 2 sites per region compared to 1 lead to higher identification of p-syn?*

| P | Patients with suspected or confirmed synucleinopathy (i.e. Parkinson’s disease, Multiple System Atrophy, Dementia with Lewy bodies, Pure Autonomic Failure), or healthy controls. |
| --- | --- |
| I/C | indirect immunofluorescence in skin biopsy samples at 2 sites per region compared to 1 |
| O | Frequency of analysable specimens  Diagnostic accuracy indicators in detecting p-syn: sensitivity, specificity, overall accuracy, false rates  Reliability indicators in detecting p-syn: raw agreement, kappa statistic |
| Study design | - consecutive suspected patients  - patients vs healthy controls |

**TOPIC 3: SIZE OF SKIN SAMPLES TO BE ANALYZED**

*3.1 How thick should the skin section be to better assess the presence of p-syn?*

- Possible thickness: e.g., 10 vs 50 micron

*In patients with suspected synucleinopathy, does indirect immunofluorescence in skin biopsy samples perform better diagnostic accuracy by analyzing 50-micron thick skin sections compared to 10-micron in identifying p-syn?*

| P | Patients with suspected or confirmed synucleinopathy (i.e. Parkinson’s disease, Multiple System Atrophy, Dementia with Lewy bodies, Pure Autonomic Failure), or healthy controls. |
| --- | --- |
| I/C | indirect immunofluorescence in skin biopsy samples using 50-micron thick skin sections compared to 10-micron |
| O | Frequency of analysable specimens  Diagnostic accuracy indicators in detecting p-syn: sensitivity, specificity, overall accuracy, false rates  Reliability indicators in detecting p-syn: raw agreement, kappa statistic |
| Study design | - consecutive suspected patients  - patients vs healthy controls |

*3.2 Which is the diameter of the punch biopsy to use?*

- Possible punch diameter: e.g. 3 vs 5 mm

*In patients with suspected synucleinopathy, does indirect immunofluorescence in skin biopsy samples performs better diagnostic accuracy by collecting samples using skin punch biopsy of 3 mm of diameter compared to 5 mm in detecting p-syn?*

| P | Patients with suspected or confirmed synucleinopathy (i.e. Parkinson’s disease, Multiple System Atrophy, Dementia with Lewy bodies, Pure Autonomic Failure), or healthy controls. |
| --- | --- |
| I/C | indirect immunofluorescence in skin biopsy samples using skin punch biopsy of 3 mm of diameter compared to 5 mm |
| O | Frequency of analysable specimens  Diagnostic accuracy indicators in detecting p-syn: sensitivity, specificity, overall accuracy, false rates  Reliability indicators in detecting p-syn: raw agreement, kappa statistic |
| Study design | - consecutive suspected patients  - patients vs healthy controls |

**TOPIC 4: QUANTITATIVE MEASURE OF MISFOLDED α-SYNUCLEIN IN SKIN SAMPLES**

*4.1 Which analysis method for p-syn quantification is the most reliable and correlated to the clinical involvement?*

- Distribution coefficient (DS) vs sample’s rate (SR) vs fiber’s rate (FR) vs number of positive fibers (NP)

*In patients with suspected synucleinopathy, does indirect immunofluorescence for p-syn in skin biopsy samples present a more reliable quantification by the DS compared to SR or FR or NP which lead to a better correlation with clinical involvement?*

| P | Patients with suspected or confirmed synucleinopathy (i.e. Parkinson’s disease, Multiple System Atrophy, Dementia with Lewy bodies, Pure Autonomic Failure), or healthy controls. |
| --- | --- |
| I/C | indirect immunofluorescence in skin biopsy samples using quantification by the DS compared to SR or FR or NP |
| O | a more reliable quantification  a better correlation with clinical involvement |
| Study design | - consecutive suspected patients  - patients vs healthy controls |

**TOPIC 5: SPECIFIC MARKER OF SKIN *α-SYNUCLEIN* TO IDENTIFY A SYNUCLEINOPATHY**

*5.1 Which form of α-synuclein in skin samples best represents an underlying pathology related to synucleinopathies?*

- e.g. p-syn vs native (n-syn) vs oligomer

*In patients with suspected synucleinopathy, does indirect immunofluorescence of skin biopsy samples for specific types of misfolded α-synuclein (e.g., p-syn, oligomer) compared to native synuclein (n-syn) lead to better diagnostic accuracy in identifying a pathological form of synuclein?*

| P | Patients with suspected or confirmed synucleinopathy (i.e. Parkinson’s disease, Multiple System Atrophy, Dementia with Lewy bodies, Pure Autonomic Failure), or healthy controls. |
| --- | --- |
| I/C | indirect immunofluorescence in skin biopsy samples for specific types of misfolded α-synuclein (e.g., p-syn, oligomer) compared to native synuclein (n-syn) |
| O | Frequency of analysable specimens  Diagnostic accuracy indicators in detecting p-syn: sensitivity, specificity, overall accuracy, false rates  Reliability indicators in detecting p-syn: raw agreement, kappa statistic |
| Study design | - consecutive suspected patients  - patients vs healthy controls |

**TOPIC 6: SAFETY OF THE SKIN BIOPSY**

*6.1 What are the safety issues of skin biopsy in the population of people with suspected synucleinopathies?*

- e.g., *type of adverse events,* *risk factors*

*In patients with suspected synucleinopathy, does skin biopsy comparing the different techniques lead to significant health risks?*

| P | Patients with suspected or confirmed synucleinopathy (i.e. Parkinson’s disease, Multiple System Atrophy, Dementia with Lewy bodies, Pure Autonomic Failure), or healthy controls.  Subgroup of risks in terms of comorbidities |
| --- | --- |
| I/C | Skin biopsy comparing sites and number of samples, and size of the sample |
| O | Type of adverse events |
| Study design | - consecutive suspected patients  - patients vs healthy controls |
