# Supplementary material for: Standardized protocol to detect phosphorylated α-synuclein in skin biopsies: recommendations from an expert panel
Source: Mol Neurodegener. 2026 Jun 25;21:54. doi: 10.1186/s13024-026-00966-w (PMC13560214; doi:10.1186/s13024-026-00966-w)
Supplement: Supplementary file 2 [file 13024_2026_966_MOESM2_ESM.docx]

**Identification of studies**

Records removed *before screening*:

Duplicate records (n = 531)

Language other than English (n = 8)

Records identified from:

PUBMED (n = 501)

SCOPUS (n = 763)

**Identification**

Records excluded:

(n = 24)

- Not original studies

Records screened

(n = 725)

Reports sought for retrieval

(n = 701)

Reports not retrieved

(n = 0)

**Screening**

Reports assessed for eligibility

(n = 701)

Reports excluded:

Not relevant to topic (n = 582)

Did not include required outcome measures (n = 87)

Studies included in review

(n = 32)

**Included**

Source: Page MJ, et al. BMJ 2021;372:n71. doi: 10.1136/bmj.n71.

This work is licensed under CC BY 4.0. To view a copy of this license, visit <https://creativecommons.org/licenses/by/4.0/>
